# Rapid determination of trace cadmium in drinking water using laser-induced breakdown spectroscopy coupled with chelating resin enrichment

**DOI:** 10.1038/s41598-019-46924-z

**Published:** 2019-07-18

**Authors:** Hongwu Tian, Leizi Jiao, Daming Dong

**Affiliations:** 1 College of Information and Electrical Engineering, Shenyang Agricultural University, Shenyang, 110866 China; 2 Beijing Research Center of Intelligent Equipment for Agriculture, Beijing Academy of Agriculture and Forestry Sciences, Beijing, 100097 China

**Keywords:** Natural hazards, Fluorescence resonance energy transfer

## Abstract

The determination of heavy metals in drinking water is of great importance, but it is hard to realize rapid and *in-situ* measurement. Laser-induced breakdown spectroscopy is an effective method for both solid and liquid sample analysis with advantages of fast and micro-destructive. However, the concentrations of heavy metals in drinking water is too low to be directly detected using LIBS. In this study, we enhanced the sensitivity of LIBS by coupling with chelating resin, which is usually used for water purification. The resin provided a rapid enrichment of the heavy metal, so the limits of detection of common LIBS system was much enhanced. Using Cadmium as the representative heavy metal, PLSR model for predicting Cd were built based on the spectral intensity (Cd 214.4 nm) with concentrations from 0 to 100 µg/L, and resulted in correlation coefficient of 0.94433 and RMSE of 7.1517 µg/L. The LoD was 3.6 µg/L. Furthermore, the volume, resin mass, adsorption time, and LIBS system parameters were optimized for practical applications. We also demonstrated that the resin can be recycled without loss in sensing ability. The combination of chelating resin with LIBS provides inexpensive, rapid, and sensitive detection method of trace heavy metal contaminants in drinking water.

## Introduction

With the rapid development of modern industry and agriculture, heavy metals have become important micropollutants in the environment^1,2^. Industrial production and pesticide and fertilizer use generate large volumes of wastewater containing heavy metals, which can contaminate surface water and ground water through runoff and leaching. Heavy metals are not biodegradable, undergo bioenrichment, and have high toxicity, with even small doses having the potential to cause serious human health problems through the food chain^3–5^. Consequently, heavy metals are one of the most dangerous pollutants in water.

The Food and Agricultural Organization, US Environmental Protection Agency, and World Health Organization have set permissible limits for different heavy metals in drinking water^6–8^. Among the heavy metals, cadmium is very hazardous and the US Environmental Protection Agency has proposed a limit of 0.005 mg/L. Methods for detection of heavy metals at part per billion levels in drinking water are required. Standard procedures involve sample collection and preparation before UV spectrophotometry, liquid chromatography, or atomic absorption spectroscopy analysis^9–12^. These methods have high sensitivity and accuracy, but they are time-consuming and some of the methods may cause secondary pollution^13,14^. In recent years, new analytical methods, such as inductively coupled plasma-optical emission spectrometry^15,16^, X-ray fluorescence spectrometry^17,18^, and neutron activation analysis^19^, have also been used for detection of heavy metals in drinking water. However, these methods also have disadvantages, such as requiring expensive equipment and complex sample preparation, and they cannot be used for rapid, on-site measurements of drinking water quality. Therefore, a rapid and inexpensive method is required for on-site analysis of heavy metals, and development of portable devices.

Laser-induced breakdown spectroscopy (LIBS) is a spectrochemical analysis method based on spectra plasmas that generated by pulsed lasers, it is a promising optical detection and analytical technology that has been widely used in industrial, environmental, and geological analyses, farmland soil analysis, nanomaterials analysis, space and planetary exploration, and other fields with its advantages of nondestructive, rapid and simulataneous multi-elemental analysis^20,21^. Liquid analytical methods based on LIBS can be conducted using one of the following methods: (1) immersion of a fiber into the liquid to be measured, excitation of the plasma in the liquid, and then collection of the emission spectrum^22,23^; (2) conversion of the bulk liquid sample into droplets, mist, or a fluid jet stream, and then breaking down of the sample with a laser for analysis^24–28^; and (3) transformation of the liquid into a solid sample by freezing^29^ or by filtration through a filter paper, by ultrafiltration, or through an ion exchange membrane^30–34^. However, all of these methods cannot be used for the measurement of heavy metals in drinking water because they are inconvenient to operate and the detection capability cannot reach part per billion levels.

Chelating resins^35^ are often used for water purification because their macroporous skeletons and carboxyl functional groups can remove heavy metal ions from aqueous solutions through physical adsorption and ion exchange based on the selectivity for ions with different valences. This feature improves the ability of LIBS for heavy metal detection.

In the present study, a new method using LIBS coupled with chelating resin was used to measure heavy metals in drinking water. To the best of our knowledge, this is the first study to enhance LIBS signals using chelating resin. The main purposes of this study were: (1) to enrich Cd in drinking water using chelating resin and verify its effect on improving the detection ability of LIBS for heavy metal measurements in liquids; (2) to verify the quantitative analysis capability of the new method using LIBS and chelating resin; and (3) to evaluate the influence of detection limits and experimental parameters on quantitative analysis, and propose recommended parameters for practical application.

## Results

### Effect of chelating resin on the enrichment of Cd

**Figure 1:**
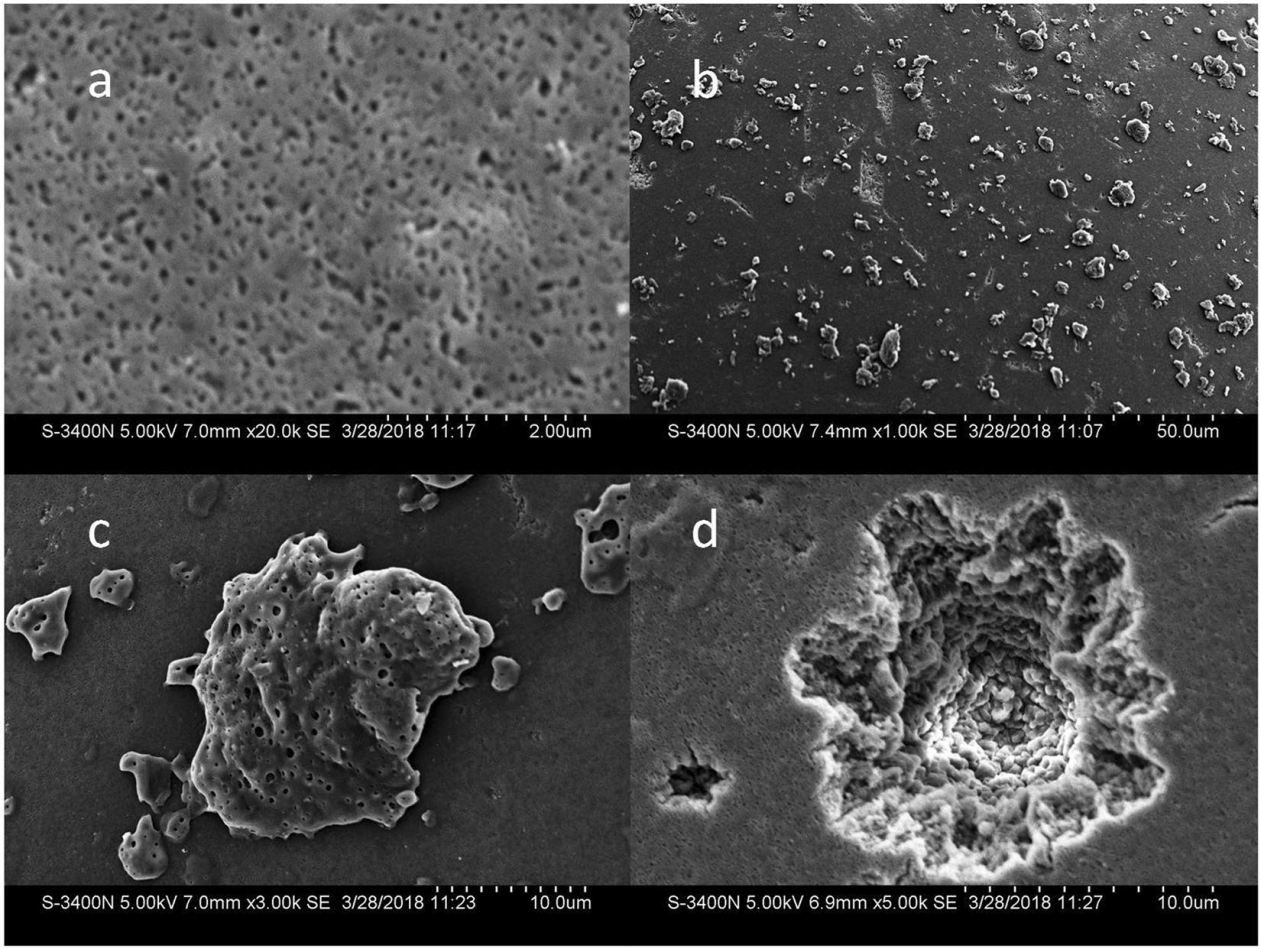
Comparison of SEM images of ECS60 before and after enrichment of Cd. (**a**) ECS60 surface pore structure; (**b**) ECS60 surface structure after absorption of 100 µg/L Cd(NO_3_)_2_ solution; (**c**) Chelated crystal on ECS60 after chelating; (**d**) Laser ablating pit on ECS60 surface.

The chelating resin ECS60 used in our work is a sodium Niminodiacetic acid chelating resin that contains opaque particles with diameters between 0.3 and 1.2 mm. Scanning electron microscopy(SEM) of its surface showed it was rough and contained many pores (Fig. 1a) because of its styrene skeleton. Heavy metal ions are attracted to the dicarboxylic ions and exchange with Na ions, which removes the metal ions from water.

By contrast, after absorption of 500 mL of the (Cd(NO_3_)_2_) solution (100 µg/L), irregular crystals protrusion formed by chelation were observed on the surface of resin (Fig. 1b,c). Laser will ablate the chelate crystals as well as the surface of resin, pits were observed on the resin surface (Fig. 1d). These results showed that the ECS60 resin could adsorbed Cd by chelating effect and make chelating crystals adhere to its surface.

### Enhancement of LIBS signals from heavy metals in drinking water

**Figure 2:**
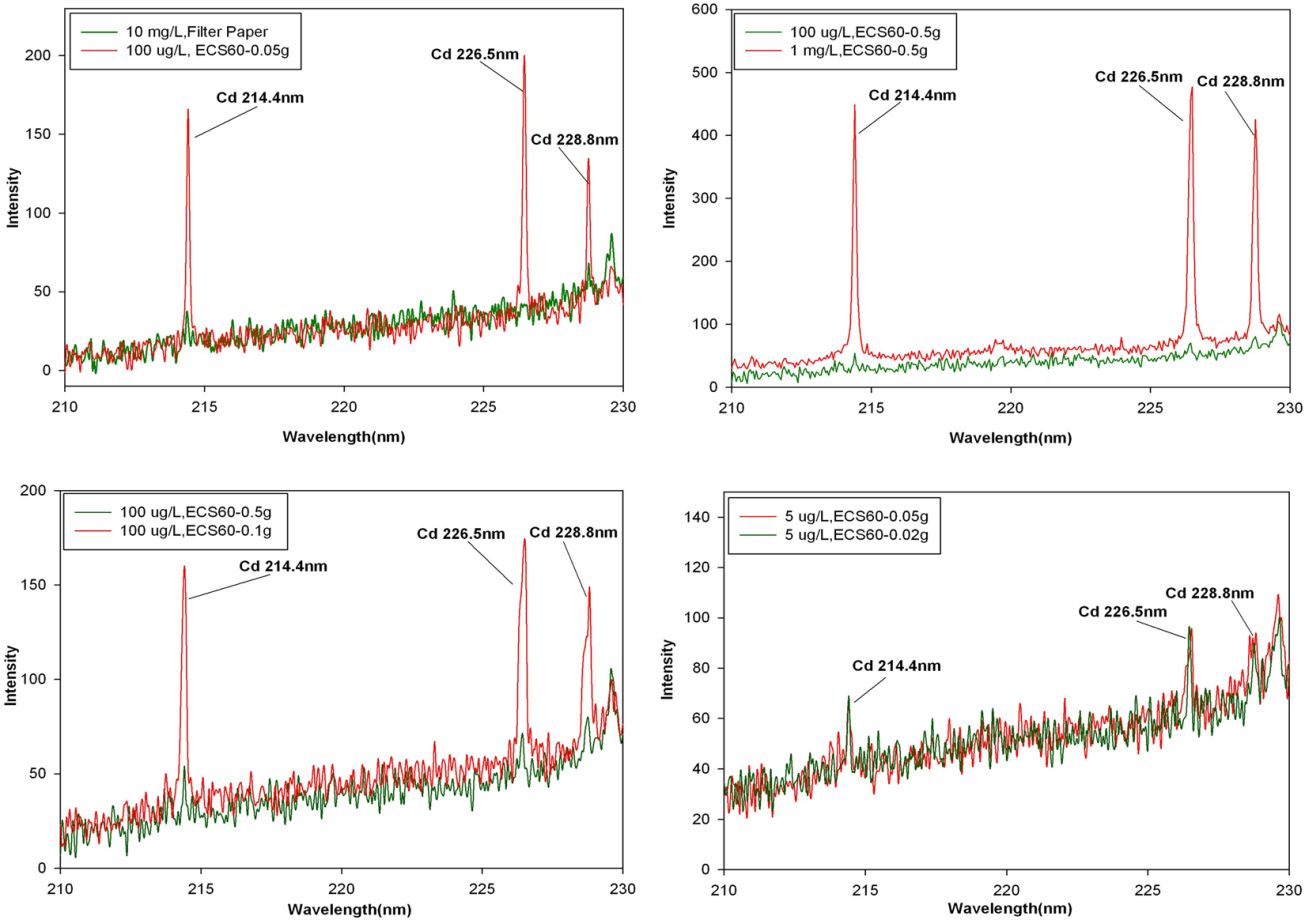
Spectrums under different experimental conditions. (**a**) Comparison of spectral intensity for filter paper enrichment and resin enrichment for Cd; (**b**) comparison of LIBS signals intensity of the Cd concentration 1 mg/L and 100 µg/L under 0.5 g adsorbent condition; (**c**) comparison of LIBS signals intensity of the adsorbent mass 0.5 g and 0.1 g at a Cd concentration of 100 µg/L; (**d**) comparison of LIBS signals intensity of the adsorbent mass 0.05 g and 0.02 g on condition that Cd concentration was 5 µg/L.

To investigate the effect of resin enrichment of the heavy metal on the LIBS signal, we recorded LIBS signals for the (Cd(NO_3_)_2_) solution after enrichment with filter paper and with the resin. The spectral intensities for enrichment of a 10 mg/L solution using filter paper and a 100 µg/L solution using the resin were compared (Fig. 2a). The concentrations of heavy metal differed by 100 factor, whereas the spectral intensity at 214.4 nm after resin enrichment was much higher than that after filer paper enrichment, with no peak observed in this region after filter paper enrichment. Therefore, the ECS60 resin provides good enrichment of Cd in water, which should improve detection by LIBS.

### Comparison of the enrichment effects with different experimental parameters

First, we compared spectra obtained with the same mass of resin and different concentrations of (Cd(NO_3_)_2_). For convenience, we used 500 mL of Cd(NO_3_)_2_ solution and 0.5 g of ECS60. Characteristic peaks were detected with 1 mg/L (Cd(NO_3_)_2_), but not with 100 µg/L (Cd(NO_3_)_2_) (Fig. 2b). It can be concluded that concentration of Cd is an important factor which effect the spectral intensity under the same condition of resin mass.

Compared with the relevant limits for Cd in China, the above Cd detection capability with 0.5 g of resin (100 µg/L Cd(NO_3_)_2_) would meet the standard for industrial wastewater (GB 8978-1996) but not that for drinking water (5 µg/L, GB 5749-2006). Consequently, we investigated changing the mass of resin and maintaining the concentration of the Cd(NO_3_)_2_ solution. We decreased the mass of ECS60 resin from 0.5 g to 0.1 g, and repeated the experiment with 100 µg/L (Cd(NO_3_)_2_). Characteristic peaks for Cd were observed at 214.4 nm, 226.5 nm and 228.8 nm (Fig. 2c), and the spectral intensities were nearly four times those obtained with 0.5 g of the resin. This occurred because the amount of Cd adsorbed on each particle of the resin increased as the mass of resin decreased.

Based on these results, experiments were performed with lower masses of resin to determine if lower concentrations of Cd could be detected. With 0.05 g of resin, characteristic peaks were observed in the spectrum with 5 µg/L (Cd(NO_3_)_2_) (Fig. 2d). However, when the resin mass was reduced to 0.02 g, the spectrum was almost the same as that recorded with 0.05 g of resin. Therefore, reducing the resin mass will increase the intensities of characteristic peaks only up to the point where the resin becomes saturated.

**Figure 3:**
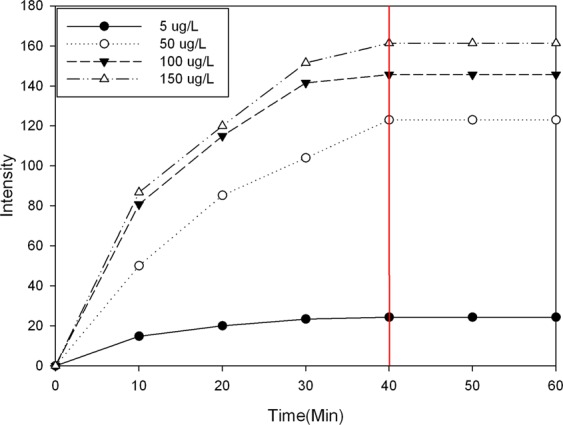
The adsorption effect changing trend at four concentrations.

Finally, we studied the effect of the adsorption time on the spectra. In practical applications, the mass of adsorbent and adsorption time are important parameters in determining whether a method can be used for rapid detection. Based on the previous results, we used a resin mass of 0.05 g, solution volume of 500 mL, and four (Cd(NO_3_)_2_) concentrations (5 µg/L, 50 µg/L, 100 µg/L, and 150 µg/L). The effect of the adsorption time was evaluated (Fig. 3). When the adsorption time reached 40 min, the spectral intensity stabilized at all concentrations. The spectral intensity for the 150 µg/L solution was much higher than that for the 100 µg/L solution, which indicated that the adsorption did not reach saturation for the 100 µg/L solution with 0.05 g of resin. Thus, a solution volume of 500 mL, adsorption time of 40 min, and resin mass of 0.05 g are recommended.

### Quantitative analysis of Cd in drinking water

Based on our results and the national standards for Cd in drinking water and industrial wastewater, we performed quantitative analysis for Cd in solutions with concentrations ranging from 0 to 100 µg/L.

To reduce experimental error, the original spectra were pre-treated before they were used for quantitative analysis. In our study, abnormal data could arise from laser energy fluctuations or differences in the adsorption uniformity. These data were deleted, and then the spectral data measured at different concentrations were averaged.

We quantitatively analyzed the relationship between the Cd concentration and the spectral intensity at 214.4 nm, and established a calibration curve using 9 water samples spiked with (Cd(NO_3_)_2_) (0 µg/L, 5 µg/L, 10 µg/L, 30 µg/L, 40 µg/L, 60 µg/L, 70 µg/L, 90 µg/L, and 100 µg/L).

**Figure 4:**
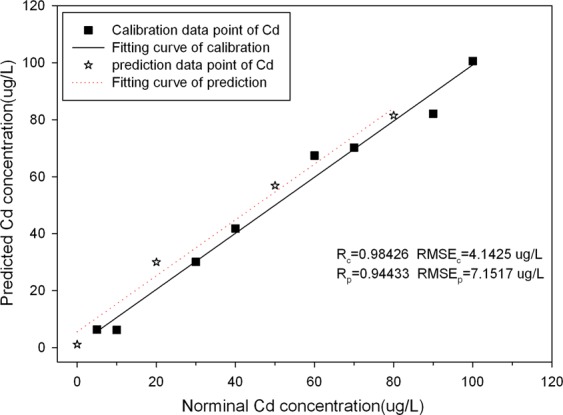
Calibration and prediction results of PLSR model using spectral data at Cd 214.4 nm.

Three concentration including 20 µg/L,50 µg/L and 80 µg/L (marked as sample 1#, 2# and 3#) were selected as prediction samples, and the other gradient data of concentration as calibration samples. The spectral intensity and the corresponding Cd concentration were used as model inputs, PLRS quantitative analysis model of Cd was built. Figure 4 showed the calibration curve of the modelling and prediction results of PLSR, the correlation coefficients (Rc and Rp) for modelling and prediction were 0.98426 and 0.94433 respectively, and it showed obvious linear relationship between the measurement value and actual value of Cd concentration.

Meanwhile, Table 1 showed the analytical results of PLSR for prediction Cd content. The relative error gradually decreased as the Cd concentration increased, and relative error of sample 2# and 3# were much lower than that of sample 1#, it was probably caused by the parameter error of the LIBS system and inhomogeneous distribution of the chelate on the surface of resin under low Cd concentration.

**Table 1:** Analytical results of PLSR for predicting Cd content.

Correlation coefficient of prediction	Verification samples	Predicted content (µg/L)	Nominal Content (µg/L)	Absolute Error (µg/L)	Relative Error (%)
0.94433	1#	30.018	20	10.018	33.37
	2#	56.908	50	6.908	12.14
	3#	81.489	80	1.489	1.83

The LOD was calculated as LOD = 3σ/S, where σ is the standard deviation of background spectrum and S is the slope of the calibration curve, and was 3.6 µg/L for Cd. With the aid of high efficacy of enrichment effect of ECS60, our method has largely improved the LOD of Cd in water, compared with other conventional methods, as showed in Table 2. Although, the LOD of Cd can reach 29 µg/L using graphite groove, the experimental system is more complex at the same time^36^. The experimental results proved that the chelation between resin and heavy metals is much effective than other traditional enrichment methods, and no other equipment is required to participate. This method is simple and sensitive enough to meet the needs of national standard for drinking water quality (5 µg/L).

**Table 2:** The LODs of Cd element obtained by different methods.

LODs	Matrix	Method	References
7.1 mg/L	water	Direct detection	^22^
1.4 mg/L	ice	Liquid-solid transformation	^22^
0.21 µg/mL	ion exchange membranes	Filtering and Enrichment	^38^
29 µg/L	graphite groove	Heating and Enrichment	^36^

Our results above showed that different resin masses, solution volumes, solution concentrations, and enrichment times affected the Cd peak intensities in LIBS. These parameters can be adjusted to achieve quantitative analysis of Cd in different concentration ranges. For example, decreasing the resin mass can reduce the LOD but may compress the quantitative analysis range because of adsorption saturation. Conversely, increasing the resin mass can extend the upper limit of the range, but this is at the expense of the LOD. It can be concluded that the amount of resin is a key factor for establishing the calibration curve. The amount of resin can be determined experimentally, and then new model can be established for different concentration ranges based on different water samples. It is also feasible to cope with very low or very high concentration by increasing the sample amount or diluting the water sample without changing the resin amount. In consideration of application requirements, we recommend a solution volume of 500 mL, resin mass of 0.05 g, and concentration range from 0 to 100 µg/L. These parameters can meet the detection standard for Cd in drinking water and industrial wastewater.

### Regeneration and reuse of ECS60

**Figure 5:**
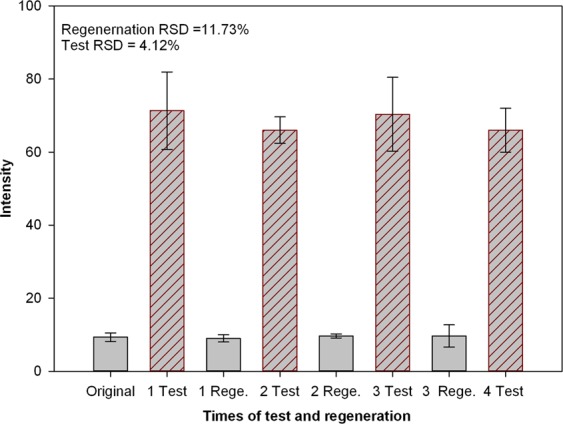
Comparison of the spectral intensity of ECS60 after three times regenerations and three times adsorptions.

ECS60 is a chelating resin that can be reused after washing with 5% HCL and activation with 5% NaOH. We tested the capability of ECS60 in repetitive measurements after regeneration. The resin was tested before use, after enrichment, and again after regeneration. The enrichment and regeneration steps and measurements were repeated for three times (Fig. 5). The characteristic peak for Cd at 214.4 nm was not observed after regeneration of the resin. After reuse of the resin in a 50 µg/L (Cd(NO_3_)_2_) solution, the characteristic peak was easily detected. Therefore, ECS60 can be reused after regeneration and activation, which will reduce material costs in practical applications. However, this process uses chemical reagents.

## Discussion

The chelating resin ECS60 was used as an absorbent, and its effect on enrichment of Cd in drinking water and enhancement of LIBS signals was investigated. A comparison of filter paper and ECS60 for enrichment confirmed that ECS60 was more effective than filter paper at enriching Cd. Next, we evaluated the impact the resin mass, solution concentration, and adsorption time on the enrichment. The optimum parameters were a resin mass of 0.05 g, sample volume of 500 mL, and adsorption time of 40 min. The characteristic peak for Cd at 214.4 nm was selected, and a regression model based on the peak intensity and concentration of Cd (0~100 µg/L) was established. The predictive correlation coefficient (Rp) of PLSR was 0.94433, and the LOD was 3.6 µg/L, which was lower than the limit set by standards in China for drinking water. Furthermore, the activation, regeneration and recycling capability of ECS60 were verified. These results show that ECS60 can greatly improve the detection capability of LIBS for Cd in drinking water. This method could be used for monitoring drinking water quality and quantitative analysis, and ECS60 is safe and low costs without leaching risk in during use thanks to its stability of physicochemical properties in acidic, alkaline and organic solvents. The LIBS method combined with the chelating resin could be applied to rapid and quantitative measurement of other trace heavy metal elements in drinking water.

## Materials and Methods

### Materials

Chelating resin ECS60^37^ was selected as the enrichment matrix. ECS60 has a styrene skeleton with iminodiacetic acid functional groups and a macroporous structure. It removes heavy metal cations from aqueous solutions by chelation via attraction of metal ions to dicarboxyl groups and nitrogen atom electron donation. Furthermore, the chelation is selective with an order for different metals of Cu > Pb > Ni > Zn > Co > Cd > Fe(II) > Mn, which means the chelating resin has different chelation efficiency for different heavy metal ions.

The contaminated water sample used in this study was prepared by dissolving cadmium nitrate (Cd(NO_3_)_2_) crystals in deionized water.

### Experimental equipment

LIBS system used in this study contained a Q-Switched Nd: YAG laser (Dawa-200, Beamtech Optronics Co., Ltd., Beijing, China), a spectrometer, a signal delay generator, and a three-dimensional moving platform. The fundamental frequency of the laser was 1064 nm, the maximum output energy was 200 mJ, the maximum repetition frequency was 20 Hz, the pulse width was 3~5 ns, and the exit angle was within 1 mrad. The laser beam was reflected by the beam splitter and then focused on the top of the resin sphere. The laser-induced plasma signal was collected by an optical fiber and transmitted to the spectrometer. The spectrometer (HR2000+, Ocean Optics) had a spectral range of 200∼1100 nm, resolution of 0.2 nm, and signal-to-noise ratio of 250:1. A three-dimensional moving platform with a stepper motor to control axial movement in three directions was used to obtain measurements at different positions in the sample.

### Experimental design

Excessive laser energy would break the resin sphere, and we set the output energy of the laser to 120 mJ for these experiments. The spectral range of the spectrometer was set to 200~1000 nm, and the integration time was set to 2 ms. We focused on the atomic emission spectroscopy of Cd. Based on previous experiments, the detector delay time to the laser was set at 0.5 µs. Before the experiments, the background spectrum was measured, and later used for correction.

**Figure 6:**
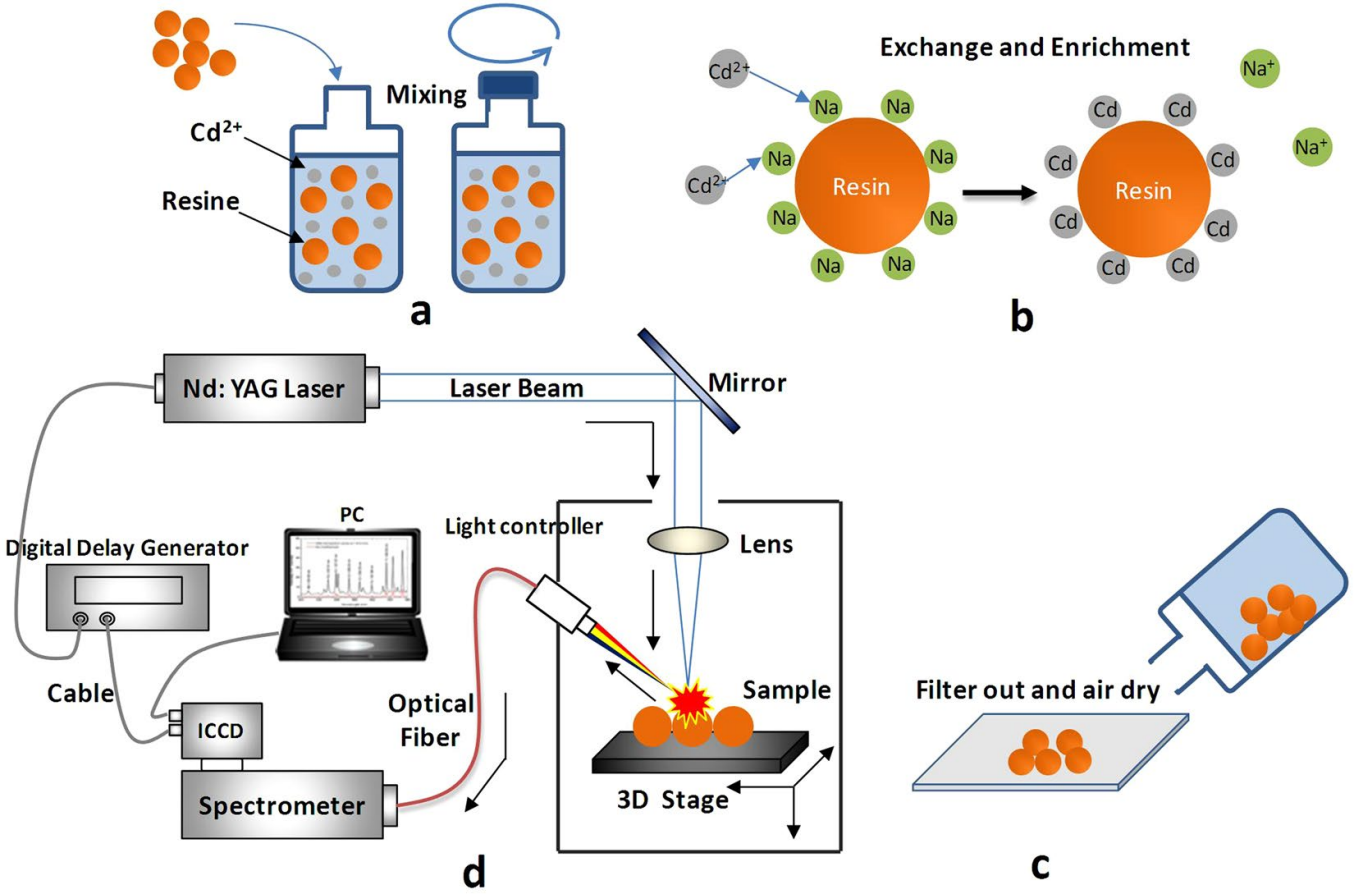
The main steps of the experiment. (**a**) Sample mixing and shaking; (**b**) Ion exchanging reaction and Cd enrichment; (**c**) Filtering and drying of the resin; (**d**) The structure of LIBS experimental system.

Experiments were conducted inside at 23 °C and standard atmospheric pressure. First, 50 g of ECS60 was weighed, washed with purified water, filtered, and dried in the air for 10 min (temperature = 23 °C). Then, 11 samples of ECS60 (0.05 g) were weighed separately, placed in bottles, and mixed with (Cd(NO_3_)_2_) solutions with a concentration gradient over the 11 bottles. The volume of solution added to each bottle was 500 mL. All the bottles were shaken for 40 min to ensure that the ECS60 fully mixed with the solution. Next, 10∼15 resin particles were isolated from each bottle, placed on a glass slide with double-sided tape, and examined. The glass slides were placed on a sample stage, and the focus was adjusted using a CCD so that the top surface of the resin sphere was located exactly at the focal point of the laser focusing system. Each time a sphere was selected for measurement, the sample concentration was measured five times, and the average value was taken for each spectrum. Figure 6 shows the experimental procedure.
